# Pulse Oximeter Oxygen Saturation in Prediction of Arterial Oxygen Saturation in Liver Transplant Candidates

**DOI:** 10.5812/hepatmon.15449

**Published:** 2014-04-07

**Authors:** Seiyed Mohammad Ali Ghayumi, Abolfazl Khalafi-Nezhad, Zahra Jowkar

**Affiliations:** 1 Department of Internal Medicine, Shiraz University of Medical Sciences, Shiraz, IR Iran; 2 Department of Operative Dentistry, School of Dentistry, Shiraz University of Medical Sciences, Shiraz, IR Iran

**Keywords:** Blood Gas Monitoring, Transcutaneous, Liver Cirrhosis, Oximetry, hypoxemia, Liver Transplantation

## Abstract

**Background:**

Liver transplant is the only definitive treatment for many patients with end stage liver disease. Presence and severity of preoperative pulmonary disease directly affect the rate of postoperative complications of the liver transplantation. Arterial blood gas (ABG) measurement, performed in many transplant centers, is considered as a traditional method to diagnose hypoxemia. Because ABG measurement is invasive and painful, pulse oximetry, a bedside, noninvasive and inexpensive technique, has been recommended as an alternative source for the ABG measurement.

**Objectives:**

The aim of this study was to evaluate the efficacy of pulse oximetry as a screening tool in hypoxemia detection in liver transplant candidates and to compare the results with ABGs.

**Patients and Methods:**

Three hundred and ninety transplant candidates (237 males and 153 females) participated in this study. Arterial blood gas oxyhemoglobin saturation (SaO_2_) was recorded and compared with pulse oximetry oxyhemoglobin saturation (SpO_2_) results for each participants. The area under the curve (AUC) of receiver operating characteristic (ROC) curves was calculated by means of nonparametric methods to evaluate the efficacy of pulse oximetry to detect hypoxemia.

**Results:**

Roc-derived SpO_2_ threshold of ≤ 94% can predict hypoxemia (PaO_2 _< 60 mmHg) with a sensitivity of 100% and a specificity of 95%. Furthermore, there are associations between the ROC-derived SpO_2_ threshold of ≤ 97% and detection of hypoxemia (PaO_2 _< 70 mmHg) with a sensitivity of 100% and a specificity of 46%. The accuracy of pulse oximetry was not affected by the severity of liver disease in detection of hypoxemia.

**Conclusions:**

Provided that SpO_2_ is equal to or greater than 94%, attained from pulse oximetry can be used as a reliable and accurate substitute for the ABG measurements to evaluate hypoxemia in patients with end stage liver disease.

## 1. Background

Up to now, liver transplant is the only definitive treatment for many patients with end stage liver disease (1). A variety of factors such as cardiovascular and respiratory diseases can affect the success rate of the liver transplant surgery (2, 3). Moreover, it has been reported that the presence and severity of preoperative pulmonary diseases directly affect the rate of postoperative complications of liver transplantation (4). One of the most common disorders of pulmonary vasculature resulting in pulmonary dysfunction among liver transplant candidates is hepatopulmonary syndrome (HPS) (5). Abnormal arterial gas exchanges caused by intra pulmonary vasodilatation, the resulting hypoxemia and/or widened alveolar-arterial oxygen gradient are HPS characteristics (6, 7). HPS prevalence has been reported as 16% to 33% and 10-17% in liver transplant candidates with end stage liver disease and patients with cirrhosis, respectively (8-14). Ghayumi showed that hypoxemia in cirrhotic candidates for liver transplantation had prevalence of 14%, without significant change over the time (15, 16). The presence of HPS increases post liver transplant mortality rate, although liver transplant is the only effective treatment for HPS (9, 17). It has been suggested that the presence of preoperative arterial oxygen pressure (PaO_2_) < 50 mm Hg is associated with poor post-transplant prognosis (18). Thereby, screening for HPS is an essential preliminary in all liver transplant candidates (6). The Arterial blood gas (ABG) measurement, performed in many transplant centers, is considered as a traditional method to detect hypoxemia caused by HPS (19). Due to invasive and painful nature and possible side effects of ABGs such as hematoma, infection, aneurysm and fistula formation, some efforts have been made to introduce an alternative method (20-23). In this context, pulse oximetry, a bedside, noninvasive and inexpensive technique, has been evaluated as an alternative source in some studies (24). According to a study by Abrams, pulse oximetry oxyhemoglobin saturation (SpO_2_) 97% or less was an indicator for mild hypoxemia (PaO_2 _< 80 mm Hg), whereas PaO_2_ less than 60 mmHg in all patients could be identified by SpO_2_ 94% or less (24). Similarly, Argueddas found pulse oximetry as a reliable predictor to detect the presence and severity of hypoxemia in patients with hepatopulmonary syndrome (25).

## 2. Objectives

Due to the controversial results and few numbers of studies about the establishment of pulse oximetry as a screening tool to identify hypoxemia in liver transplant candidates, the aim of this study was to evaluate the efficacy of this method to detect hypoxemia in liver transplant candidates and to compare the results with ABGs.

## 3. Patients and Methods

This was a cohort prospective study with 390 participants (237 males and 153 females). Participants were recruited from patients referred to Shiraz Transplant Center, Shiraz, Iran for preoperative liver transplant evaluation over 24 months (January 2010 to December 2012). All patients signed an informed consent, approved by the Ethical Committee of Shiraz University of Medical Sciences. Cirrhosis was diagnosed based on the histological findings or the combination of radiologic, clinical and laboratory evaluations. Physical examination, pulse oximetry, chest X-ray, pulmonary function test and simultaneous arterial blood gas measurements were checked as the respiratory function indicators for all the participants. In addition, Model for End-Stage Liver Disease (MELD) score (26) was used to determine patients' hepatic cirrhosis grade. Each participant was asked to sit for 15 minutes after which a finger pulse oximeter device (Oxypleth 520A Pulse Oximeter, Novametrics, Respironics Inc., Murrysville, PA, USA) was placed on the right forefinger to measure oxygen saturation in room air. Stable oxygen saturation value was recorded after one minute of pulse oximetry as SpO_2_. While patients were sitting; ABG measurements (oxy hemoglobin saturation, arterial O_2_ pressure, pH and bicarbonate level) from radial arteries were performed in room air. The mean difference between SpO_2_ and arterial blood gas oxyhemoglobin saturation (SaO_2_), considered as the bias, was calculated to determine the overestimation or underestimation of the values obtained from pulse oximetry compared to ABG measurements.

Patients were divided into two groups according to the MELD score to investigate the specificity and sensitivity of pulse oximetry to detect hypoxemia based on the severity of liver disease as follows; 1) patients with MELD score below 20, and 2) patients with MELD score equal to or greater than 20. SPSS version 18 for windows (SPSS Inc., Chicago, IL, USA) was used for data analysis. To evaluate the efficacy of pulse oximetry to detect hypoxemia in liver transplant candidates, the area under the curve (AUC) of receiver operating characteristic (ROC) curves was calculated by means of nonparametric methods. Quantitative and qualitative measurements were expressed as mean ± SD and proportions, respectively. P value less than 0.05 was considered as statistical significant. Student’s unpaired t-test and chi-square test or Fisher’s exact test was used to detect group differences and categorical variable differences, respectively. Non-parametric analysis (Spearman’s correlation test) was used to assess the association between bias values and other patient’s data.

## 4. Results

This study consisted of 390 participants as seen in Table 1, including 237 (70%) males and 153 (30%) females. The mean age of the participants was 43.5 ± 13.7. The most common causes of liver disease were cryptogenic (25.4%) and hepatitis B (23.3%). The average MELD score of the study was 18.3 ± 5.2. Abnormal chest x-ray findings were seen in 80 patients (20.8%) including elevated diaphragm in 40 patients (10.3%), pleural effusion in 28 patients (7.2%) and other types of abnormalities in 12 patients (3.1%). In pulmonary function tests, obstructive lung disease, considered as forced expiratory volume in 1 second/forced vital capacity < 70%, was found in 29 patients (7.4%) (Table 1). 

The mean MELD scores were 19 ± 5.1 and 18.3 ± 5.2 for males and females, respectively. An overestimation in pulse oximetry was seen in 63.8% of the patients, and the mean bias was 1.48 % ± 2.85 % (ranged -7% to 14%) (Table 2). Data statistical analysis revealed mean bias over 4% or less than -4% in 12.1% (n = 47) of cases. Comparing several parameters to find potential contributory factors to the bias 4 revealed that there were no statistically significant differences between the bias 4 and chest x-ray abnormalities, age, PaO_2_, MELD score, smoking, causes of liver disease, and pH. However, the marked bias was related to the PFT pattern (P = 0.018), gender (P = 0.001) and HCO_3_ (P = 0.03).

**Figure 1.**
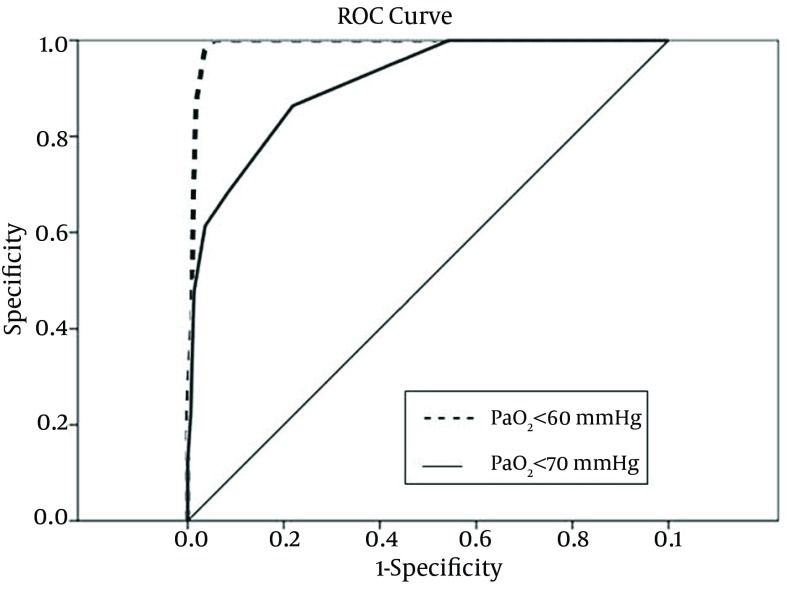
ROC Curves of SpO_2 _Measurement for the Detection of SpO_2 _< 60 mm Hg and SpO_2 _< 70 mmHg

To identify the sensitivity and specificity of SpO_2_ levels to predict hypoxemia, ROC curves were created to calculate the AUC and to determine the SpO_2_ cutoffs for detecting hypoxemia. Roc-derived SpO_2_ threshold of ≤ 94% could predict hypoxemia and a PaO_2 _< 60 mmHg with a sensitivity of 100% and a specificity of 95%, with an AUC of 0.99 (95% confidence interval (CI), 0.98-1.00) (Figure 1). Also, there were some associations between the ROC-derived SpO_2_ less than 97% and detection of hypoxemia and PaO_2 _< 70mmHg with a sensitivity of 100% and a specificity of 46%, with an AUC of 0.91 (95%CI, 0.87-1.00) (Table 3) (Figure 1). 

To detect hypoxemia (PaO_2 _< 60 mmHg) in patients with MELD score equal to or greater than 20 mmHg, ROC-derived SpO_2_ threshold of ≤ 93% had a sensitivity of 100% and a specificity of 95% with AUC of 0.98(95%CI,0.95-1.00).Based on the results of the constructed ROC curves, pulse oximetry was able to detect hypoxemia (PaO_2 _< 60 mmHg) with a threshold of ≤ 94% in patients with MELD score lesser than 20mmHg with a sensitivity of 100% and a specificity of 95% with AUC of 0.99 (95%CI, 0.98-1.00) (Table 4). 

**Table 1.** Demographics and Laboratory Data of the Patients ^a,b^

Variable	Value
**Age, y (16-81)**	43.5 ± 13.7
**Sex, Male/Female**	237/153
**MELD**	18.3 ± 5.2
**Cause of cirrhosis**	
Cryptogenic	99 (25.4)
Hepatitis B	91 (23.3)
Autoimmune hepatitis	43 (11)
Hepatitis C	40 (10.3)
Primary sclerosing cholangitis	39 (10)
Nonalcoholic fatty liver	30 (7.7)
Alcoholic cirrhosis	8 (2)
Wilson	5 (1.3)
Miscellaneous	35 (9)
**Abnormal chest x-ray**	21.6
**Pulmonary function test**	
Normal	57.4
Restrictive	33.3
Obstructive	7.5
Mixed	1
Not available	0.8

^a^ Data are presented as mean ± SD, No. (%) or %.

^b^ Abbreviations: MELD, model for End-Stage Liver Disease.

**Table 2.** ABG and Pulse Oximetry Results and Calculated Bias ^a^

Measurement	Mean ± SD	Range	95% CI
**pH**	7.46 ± 0.44	7.31-7.60	7.46-7.47
**PaO**_**2**_	77.13 ± 13.85	40-100	75.68-78.58
**PaCO**_**2**_	29.28 ± 4.85	17-46	28.7-29.7
**HCO**_**3**_	21.01 ± 3.49	10-31	20.6-21.3
**SaO**_**2**_	95.19 ± 3.22	74-99	94.8-95.5
**SpO**_**2**_	96.1 ± 2.96	80-100	95.8-96.4
**Bias**	1.48 ± 2.85	-7-14	1.20-1.77

^a^ Abbreviation: CI, confidence interval.

**Table 3.** Test Performance Characteristics of SpO_2_Measurement as Screening Tool for Hypoxemia

SpO_2_ “positive” ≤ to	PaO_2 _< 70 mm Hg, sensitivity [%]/specificity[%]	PaO_2 _< 60 mm Hg, sensitivity [%]/specificity[%]
**88%**	9/100	17/100
**89%**	12/100	23/100
**90%**	22/99.3	41/99
**91%**	36/99	67/98
**92%**	47/98	87/97
**93%**	55/97	97/96
**94%**	61/96	100/95
**95%**	68/91	100/89
**96%**	86/79	100/73
**97%**	100/46	100/41
**98%**	100/8	100/8
**99%**	100/1	100/1
**100%**	100/0	100/0

**Table 4.** Test Performance Characteristics of SpO_2 _Measurement as Screening Tool for Detection of Hypoxemia Based on the MELD Score ^a^

SpO_2_ “Positive” ≤ to	PaO_2 _< 60 mm Hg,Sensitivity [%]/Specificity[%] MELD < 20	PaO_2 _< 60 mm Hg, Sensitivity [%]/Specificity[%] MELD ≥ 20
**89%**	25/100	21/100
**90%**	37/100	47/98
**91%**	66/99	68/98
**92%**	85/98	89/97
**93%**	96/97	100/95
**94%**	100/95	100/92
**95%**	100/90	100/88
**96%**	100/72	100/74
**97%**	100/41	100/41
**98%**	100/8	100/8
**99%**	100/2	100/5
**100%**	100/0	100/0

^a^ Abbreviation: MELD, model for End-Stage Liver Disease.

## 5. Discussion

Performed to evaluate the ability of pulse oximetry to detect hypoxemia in a group of patients with end stage liver cirrhosis who referred for liver transplantation.

According to the results of this study, we could predict hypoxemia (PaO_2 _< 60 mmHg) with a sensitivity of 100% and a specificity of 95% with a SpO2 ≤ 94%. Also, with a SpO_2 _< 97%, we could detect PaO_2 _< 70mmHg with a sensitivity of 100% and a specificity of 46%. Soluble components (oxyhemoglobin) detection by transmitting a specific wavelength of light (660 to 940 nm) across a solution followed by measuring its intensity on the other side is the basis of pulse oximetry (27).

Studies on pulse oximetry accuracy have shown contradictory results. The results of this study were consistent with some previous investigations. Abrams’s study revealed that SpO_2 _equal to or below 97% is an indicator for mild hypoxemia (PaO_2 _< 80 mmHg); whereas, PaO_2_ less than 60 mmHg in all patients can be identified by SpO_2_ 94% or less (24). However, based on the results of Abrams’s study, SpO_2_ threshold of ≤ 94% can predict hypoxemia and a PaO_2 _< 70 mmHg with a sensitivity of 68% and a specificity of 99% (24). According to Argueddas, pulse oximetry was a reliable predictor to detect the presence and severity of hypoxemia in patients with hepatopulmonary syndrome. His study also showed that a threshold value for pulse oximetry of less than 94% could identify partial pressure of oxygen < 60 mmHg with a sensitivity and specificity of 100% and 93%, respectively, which is in agreement with our results. However, this study was performed in a center near the sea level. Considering the effect of altitude on SpO_2_ and PaO_2_ values, the results of this study could not be generalized to higher altitudes (25).

In contrast, some studies did not support pulse oximetry alone as a predictor of hypoxemia. According to Blasidell, pulse oximetry had poor specificity to detect hypoxemia in clinically stable patients with sickle cell anemia in outpatient setting (28). Perkins found that SpO_2_ cannot reliably detect equivalent changes in SaO_2_ by pulse oximetry in critically ill patients (29). According to a study conducted by Bourdelles in adult emergency department, pulse oximetry reduced the number of unjustified ABG determinations. However, pulse oximetry did not affect beneficial ordering of ABG measurements (30).

According to our investigation, provided that SpO_2_ is equal to or greater than 94%, pulse oximetry can be used as a substitute for ABG measurement to evaluate hypoxemia in patients with end stage liver disease. Although ABG measurement is performed in many transplant centers, it is considered as an invasive and painful procedure with possible side effects such as hematoma, infection, aneurism and fistula formation (20-23). Based on the results of this study, pulse oximetry, a simple bedside noninvasive and inexpensive low cost technique (24), can be used as a reliable alternative method to ABG measurement. Despite the benefits of pulse oximetry, some limitations such as light scattering and reflection, carboxyhemoglobin and methemoglobin, calibration, vasoconstriction and venous engorgement, motion artifact, hypothermia, hypotension, and anemia affect its accuracy (31-33). 

Other factors, such as high blood lipid concentrations, hyper alimentation, and hyper bilirubinemia, can interfere with pulse oximeter readings. Moreover, increased concentrations of bilirubin can overestimate the measured oxygen saturation. Other influencing factors on the accuracy of pulse oximetry readings are intravenous dyes, such as methylene blue, indigocarmine and indocyanine green, artificial nails and opaque nail finishes. In contrast, skin color is not considered as a limiting factor on the accuracy of pulse oximetry, which is because of using arterial pulsatile component of blood flow by the measurements and factoring out the surrounding tissue by the microcomputer (34). However, skin color was mentioned as a limiting factor in another study (35). In our study, patients were divided into two groups according to the MELD score to detect hypoxemia based on the severity of liver disease as follows; 1) patients with MELD score lesser than 20 and 2) patients with MELD score equal to or greater than 20. However, the accuracy of pulse oximetry in detecting hypoxemia was not different between the two groups.

The specificity and sensitivity of pulse oximetry to detect hypoxemia in this study were high. As a result, pulse oximetry is considered as a screening tool to detect hypoxemia in patients with liver cirrhosis candidates for liver transplantation. As mentioned above, an overestimation in pulse oximetry was present in 63.8% of the patients, and the mean bias was 1.48 % ± 2.85 % ( ranged-7% to 14%).

Although this study was performed in a relatively high altitude center (1500 meters above the sea level), the results were approximately similar to a previous study (24) performed in a center near the sea level. Thus, it seems that altitude did not affect the accuracy of pulse oximetry in detection of hypoxemia. A relatively high number of participants (390) took part in this study. Hence, it seems that the results of this study can be reliable to be applied in other liver transplant centers. However, this study was conducted on whites and the influence of race and skin color on the accuracy of pulse oximetry is still controversial and additional studies should be assigned.

According to the results of this investigation, provided that SpO_2_ is equal to or greater than 94%, pulse oximetry can be used as a reliable and accurate substitute for the ABG measurements to evaluate hypoxemia in candidates for liver transplantation. In patients with SpO_2_ values less than 94%, pulse oximetry alone is not reliable to detect hypoxemia due to overestimation of SpO_2_, so that detection of hypoxemia in those patients still requires ABG measurement. Hence, ABG measurements should be confined to those with pulse oximetry saturation less than 94%.
